# Association of Neighbourhood and Individual Social Capital, Neighbourhood Economic Deprivation and Self-Rated Health in South Africa – a Multi-Level Analysis

**DOI:** 10.1371/journal.pone.0071085

**Published:** 2013-07-29

**Authors:** Lumbwe Chola, Olufunke Alaba

**Affiliations:** 1 Health Systems and Services Research Unit, Stellenbosch University, Stellenbosch, South Africa; 2 Health Economics Unit, University of Cape Town, Cape Town, South Africa; Kenya Medical Research Institute – Wellcome Trust Research Programme, Kenya

## Abstract

**Introduction:**

Social capital is said to influence health, mostly in research undertaken in high income countries' settings. Because social capital may differ from one setting to another, it is suggested that its measurement be context specific. We examine the association of individual and neighbourhood level social capital, and neighbourhood deprivation to self-rated health using a multi-level analysis.

**Methods:**

Data are taken from the 2008 South Africa National Income Dynamic Survey. Health was self-reported on a scale from 1 (excellent) to 5 (poor). Two measures of social capital were used: individual, measured by two variables denoting trust and civic participation; and neighbourhood social capital, denoting support, association, behaviour and safety in a community.

**Results:**

Compared to males, females were less likely to report good health (Odds Ratio 0.82: Confidence Interval 0.73, 0.91). There were variations in association of individual social capital and self-rated health among the provinces. In Western Cape (1.37: 0.98, 1.91) and North West (1.39: 1.13, 1.71), trust was positively associated with reporting good health, while the reverse was true in Limpopo (0.56: 0.38, 0.84) and Free State (0.70: 0.48, 1.02). In Western Cape (0.60: 0.44, 0.82) and Mpumalanga (0.72: 0.55, 0.94), neighbourhood social capital was negatively associated with reporting good health. In North West (1.59: 1.27, 1.99) and Gauteng (1.90: 1.21, 2.97), increased neighbourhood social capital was positively associated with reporting good health.

**Conclusion:**

Our study demonstrated the importance of considering contextual factors when analysing the relationship between social capital and health. Analysis by province showed variations in the way in which social capital affected health in different contexts. Further studies should be undertaken to understand the mechanisms through which social capital impacts on health in South Africa.

## Introduction

Self-rated health has been shown to be directly related to future health status and death, with reports suggesting that people who rate their health positively are less likely to fall ill or die over the next 30 years than those who think they are not as healthy [1]. As such, self-rated health is often used as a proxy indicator of an individual's overall health status. Health status varies, not only as a result of biological factors, but also the physical and social environments [2], [3]. Housing conditions, residential areas and the work environment have all been associated with health [4], [5]. Socioeconomic circumstances such as employment, education, income and wealth are also related to health [6]. Often, people in poor living conditions report the worst health outcomes.

In recent years, there has been much discussion on the concept of social capital and its impact on health outcomes. Social capital embodies features of social organization, such as interpersonal trust, reciprocity norms, and engagements with community and neighbourhood, which achieve benefits such as improved safety and social participation [7]. Although, no single indicator can embrace the complete spectrum of social capital, there are two main domains in the literature that can be associated with the concept; the cognitive (perceived interpersonal trust, norms and reciprocity) and structural (civic participation, socializing and networking) domains that act as resources for individuals and facilitate collective action towards effective social decisions and improved outcomes [8].

Studies on the impact of social organization on health have mainly been undertaken in high income countries, showing the relationship between social capital and, among other things, depression, self-rated health and general well-being [2], [9], [10]. A few of these studies have also been conducted in low and middle income countries and very few in sub-Saharan Africa [11]–[13]. This lack of information presents a serious challenge to the expansion of the beneficial effects of social capital on health as implied in various studies [14]–[16]. It is thus very important to have evidence of social relations and their effect on health in sub-Saharan African countries, to inform decision making, resource allocation and priority setting for healthcare interventions.

It should be noted though, that the ease of building and upholding social capital depends on the political, cultural, economic and historical environment of a particular area. Therefore, differences may be observed in the levels of social capital between countries, which is often a result of differing welfare regimes [17]. Despite this, many studies assessing social capital mainly focus on individual characteristics and rarely consider group or neighbourhood characteristics of social integration in a contextual framework [18], [19]. Social capital measured at the individual level may fail to capture various group characteristics, such as neighbourhood networks and norms, which may affect health. It is thus imperative that a holistic approach is taken to studying health, because group dynamics have been shown to have a strong influence on individual health outcomes [2], [19]. We extend that it is also plausible to expect differences in the relationship between social capital and health even within a country due to contextual diversity. Therefore, the study of social capital should be context specific.

There has thus recently been a paradigm shift to assessing health effects from analysing individual level effects to group or neighbourhood level effects in multi-level analyses, which have again mainly been conducted in high income countries settings [14]–[16]. However, most studies analysing neighbourhood level effects do not control for neighbourhood level socio-economic status, which is crucial to understanding the impact of social capital on health [20]. Neighbourhood socio-economic conditions are very important for individuals' health and well-being. Better neighbourhood socio-economic status is related to higher quantity and quality of communal institutional resources and social amenities, more supportive family and neighbourhood social processes, which result in better health outcomes.

In South Africa, there has been a growing body of literature on social capital and its impact on health, addressing several issues, conceptually and methodologically. Tomita and Burns assessed the effects of neighbourhood social capital on depression, controlling for several confounders [11]. Cramm and Neiboer reported on individual social capital and its effects on self-rated health [21]. However, there is need for further insight into the relationship between social capital and health, and particularly how this relationship varies in different areas of South Africa. Thus, with the understanding that social capital is largely context specific, the aim of this paper is to examine how individual and neighbourhood level social capital, and neighbourhood deprivation relate to self-rated health using a multi-level analysis. The analysis is provided for both the overall population and further disaggregated according to South Africa's 9 provinces.

## Methods

### Study setting

South Africa is an upper-middle income country located at the southern tip of the African continent. It has a population of over 50 million people, mostly of black African ancestry [22]. The country has 9 provinces, namely: Eastern Cape, Free State, Gauteng, Kwa Zulu Natal, Limpopo, Mpumalanga, North West, Northern Cape and Western Cape. The provinces are quite distinct, each with its own legislature and provincial administration. They also have a distinctive landscape, climate, economy and population. The languages spoken in the provinces vary considerably. IsiXhosa is spoken by most people in Eastern Cape, isiZulu in Kwa Zulu Natal and Afrikaans in the Northern and Western Cape. The Northern Cape has the largest land area, but smallest population of about 1 million people, while Gauteng has the smallest land area and largest population with 12 million people (see Table 1). Gauteng also has the largest economy, contributing 33% to South Africa's gross domestic product [22].

**Table 1:** Selected socio-economic indicators by provinces, South Africa.

Province	Population	Urban population (%)	% living in formal dwelling*	Serious crime rate per 100,000	Contribution to GDP (%)	Unemploy ment rate (%)	Gini- coefficient
Western Cape	5 822 734	88.9	80.4	6601.9	14.1	23.9	0.63
Eastern Cape	6 562 053	36.6	63.2	2806.2	7.7	29.8	0.68
Northern Cape	1 145 861	70.1	82.4	3793.5	2.3	28.4	0.56
Freestate	2 745 590	68.6	81.1	4343.9	5.5	33.2	0.62
Kwazulu Natal	10 819 100	43.1	71.6	2669.9	15.8	22.5	0.77
North West	3 509 953	34.9	76.2	3061.1	6.7	23.3	0.64
Gauteng	11 328 200	97.0	79.8	4576.1	33.7	23.7	0.60
Mpumalanga	4 039 939	39.1	83.8	3073.7	7.0	29.4	0.65
Limpopo	5 404 868	11.0	89.8	1873.3	7.2	19.6	0.65
National	51 378 298	53.7	77.6	3608.7		24.9	

Sources: Stats SA (www.statssa.gov.za), *SAPS (www.saps.gov.za).

### The South African National Income Dynamics Study (SA-NIDS)

The data used in this analysis are taken from the first wave of the South African National Income Dynamics Study (SA-NIDS). This cross-sectional study was undertaken in all 9 provinces of the country in 2008, by the South African Labour and Development Research Unit (SALDRU) based at the University of Cape Town (UCT). The panel study documents the dynamic structure of household members and changes in their incomes, expenditures, assets, access to services, education, health and other dimensions of well-being. A household questionnaire and an adult questionnaire were administered to every household member aged 15 years and older. The mother or primary caregiver completed a child questionnaire for household members aged 0–14 years. The overall household response rate was 69%. The SA-NIDS provides baseline data on a sample of 28,247 individuals from 7,301 households. The sample consisted of 16,800 adults. After cleaning, the sample was 13,381. A detailed report on the SA-NIDS methodology is provided elsewhere [23].

### Measures

#### Dependent variable – Self-rated health

This was measured using a question that asked the respondent to rate their health on a scale from 1– excellent to 5– poor. Respondents were asked the following question: How would you describe your health at present? In the analysis, the variable was dichotomised into 0– poor (combining fair and poor) and 1– good (combining good, very good and excellent).

#### Demographic factors

Age was measured in single years from 15 years and included in the analysis as a continuous variable. Marital status was coded 1) never married 2) Married/cohabiting and 3) Divorced/widowed/separated. Gender was categorized as 1) male and 2) female.

#### Socio-economic factors

Education was measured in years of schooling and categorised as 1) no education, 2) primary, with 1–7 years, 3) secondary, with 8–12 years and 4) tertiary, with 13+ years of schooling. Employment included those in both formal and informal employment and was dichotomised into 1) employed and 0) unemployed. The receipt of government grants variable was created by summing the number of government grants available to individuals. This was categorised into 0) no grant, 1) receipt of grant. Household income was used as an indicator of household economic status. We used the income variable as generated in the SA-NIDS [24]. This was further categorized into deciles. We included the rural/urban dichotomy to indicate place of residence with 1) rural and 0) urban.

#### Risk factor – smoking

We controlled for smoking, which is often associated with poor health [25]. Smoking was included as a dichotomous variable indicating 0) non-smoker and 1) smoker.

#### Individual level social capital

We used civic participation and social trust as measures of individual level social capital. In the SA-NIDS, respondents were asked to indicate whether they belonged to one or more of 18 associations. We created a dichotomous variable reflecting whether 1) an individual belonged to at least 1 group or 2) did not belong to any group. Social trust was measured by a question that asked the likelihood of a neighbour returning a lost wallet containing R200, to which participants responded 1) very likely 2) somewhat likely and 3) not likely.

#### Contextual factor – Deprivation index

The deprivation index, which is a summary score of economic disadvantage, has been constructed in various studies since the 1980 s [26], with the objective of distilling a variety of deprivation measures and proxies into a single figure or index which can be used to rank areas according to intensity of deprivation [27]–[29]. In this analysis, we took the South African Index of Multiple Deprivation (SAMID) –2007 constructed at local municipality level, and linked to the SA-NIDS using the census 2001 municipality and district codes. The SAMID –2007 was constructed using the community survey of 2007 [30] across the following domains: income and material, employment, education and living environment. This aggregate measure of neighbourhood disadvantage was calculated for each local municipality and ranked from 1– most deprived to 237– least deprived municipality. The local municipality is the smallest unit of administration. A full explanation of this index can be found in Nobel et al [30]. For this study, the deprivation index was categorised into 1) most deprived, 2) moderately and 3) less deprived.

#### Contextual factor – Social capital

We measured neighbourhood social capital using four variables denoting support, association, behaviour and safety, in a summative index aggregated across households to create a neighbourhood social capital score. The variables used were respectively assessed using the following questions: 1) “How common is it that neighbours help each other out?” 2) “How common is it that neighbours do things together?” 3) “How common is it that people in your neighbourhood are aggressive?” 4) “How common is burglary and theft in your neighbourhood?” Respondents answered these questions on a scale of 1(never happens) to 5(very common). The final score ranged between 0 to 20, with a Cronbach's Alpha of 0.60. The higher the score the better the social capital.

### Data analysis

A multilevel analytical framework was used to assess factors influencing self-rated health at individual and neighbourhood/community levels. The multilevel analysis was conducted using four models as follows: Model 1 was the null, which had no variables; Model 2 included all the individual variables; Model 3 included only contextual factors; and Model 4 included all the individual and contextual variables in Models 2 and 3. Results are presented for the overall population, as well as by province. All statistical analyses were done in Stata 12 (Stata Corp. Inc. TX, USA).

## Results

Out of the total sample of 13,381, about 60% were females (Table 2). The mean age (standard deviation – SD) was 38 years (18). Most of the participants had never been married (52%), 11% were widowed or divorced and 37% were married or living as if married. The majority of respondents had secondary (56%) followed by primary (24%) education and 13% had no education. Approximately 66% of the respondents were unemployed and 36% reported that they received government grants to supplement their monthly income. The mean (SD) household income was R3,065 (R6,853). With regard to individual social capital, only about 30% of the respondents perceived their neighbours to be trustworthy (Tables 2 and 3). The province with the highest interpersonal trust level is KwaZulu Natal (23%), followed by Limpopo (14%). Approximately 64% of the respondents did not belong to any society within their communities. Civic participation was highest in KwaZulu Natal (20%) followed by Limpopo (14%), and lowest in Northern Cape (6%). The mean (SD) score for neighbourhood social capital was 13.58 (3.56). By province, the neighbourhood social capital score (SD) was highest in Limpopo 11.34 (2.89) and lowest in Gauteng 9.75 (2.96).

**Table 2:** Descriptive statistics of variables used in the analysis.

Outcome	Poor	Good	Total
Self-rated Health	(n = 2,890; 22%)	(n = 10,491; 78%)	(n = 13, 381)
**Gender**			
Male	942 (32.6)	4,439 (42.31)	5,381 (40.21)
Female	1,948 (67.4)	6,052 (57.69)	8,000 (59.79)
**Marital status**			
Married/partners	1,309 (45.29)	3,626 (34.56)	4,935 (36.88)
Widow/ divorced	696 (24.08)	809 (7.71)	1,505 (11.25)
Never married	885 (30.62)	6,056 (57.73)	6,941 (51.87)
**Age**			
15–19	92 (3.18)	2,234 (21.29)	2,326 (17.38)
20–29	245 (8.48)	2,915 (27.79)	3,160 (23.62)
30–39	352 (12.18)	1,959 (18.67)	2,311 (17.27)
40–49	543 (18.79)	1,536 (14.64)	2,079 (15.54)
50+	1,658 (57.37)	1,847 (17.61)	3,505 (26.19)
**Education**			
None	893 (30.9)	911 (8.68)	1,804 (13.48)
Primary	1,074 (37.16)	2,191 (20.88)	3,265 (24.4)
Secondary	863 (29.86)	6,677 (63.65)	7,540 (56.35)
Tertiary	60 (2.08)	712 (6.79)	772 (5.77)
**Employment**			
Unemployed	2,191 (75.81)	6,652 (63.41)	8,843 (66.09)
Employed	699 (24.19)	3,839 (36.59)	4,538 (33.91)
**Grants**			
No	2,187 (75.67)	6,254 (59.61)	8,441 (63.08)
Yes	703 (24.33)	4,237 (40.39)	4,940 (36.92)
**Residence**			
Urban	1,354 (46.85)	5,333 (50.83)	6,687 (49.97)
Rural	1,536 (53.15)	5,158 (49.17)	6,694 (50.03)
**Smoking**			
No	2,215 (76.64)	8,253 (78.67)	10,468 (78.23)
Yes	675 (23.36)	2,238 (21.33)	2,913 (21.77)
**Trust**			
No	2,050 (70.93)	7,221 (68.83)	9,271 (69.28)
Yes	840 (29.07)	3,270 (31.17)	4,110 (30.72)
**Civic participation**			
No	1,807 (62.53)	6,808 (64.89)	8,615 (64.38)
Yes	1,083 (37.47)	3,683 (35.11)	4,766 (35.62)

**Table 3:** Self-rated health and social capital measurements by province.

Province	Self-reported health status (%)		Trust (%)		Civic member ship (%)	Average Social capital
	Bad	Good	Trust	No trust		
	(n = 2,890)	(n = 10,491)	(n = 4,110)	(n = 9,271)	(n = 4,766)	n(SD)
Western Cape	11.39	14.43	13.92	13.72	13.52	10.24 (3.14)
Eastern Cape	12.69	12.79	8.33	14.64	12.88	10.55 (2.79)
Northern Cape	7.38	6.86	5.54	7.58	6.61	9.82 (3.24)
Freestate	7.41	6.49	8.41	5.96	8.07	10.59 (2.90)
Kwazulu Natal	34.85	26.92	23.44	30.81	19.94	10.57 (2.67)
North West	7.79	7.38	7.21	7.58	7.86	10.68 (3.05)
Gauteng	5.91	8.63	10.21	7.13	9.02	9.75 (2.96)
Mpumalanga	6.99	7.42	8.71	6.74	8.15	10.94 (2.91)
Limpopo	5.59	9.08	14.22	5.84	13.96	11.34 (2.89)
National	21.56	78.44	29.68	70.32	35.69	10.51 (2.93)

SD = Standard deviation.

About 78% or the respondents reported good or excellent health, and females (67%) were more likely than men to report bad health (Tables 2 and 3). When stratified by province, the highest proportion of persons reporting good health (27%) as well as bad health (35%) was in Kwazulu Natal (Table 2). The lowest proportion of persons with good health was in Freestate (6.5%) and bad health in Limpopo (5.6%). Over 50% of those reporting bad health were above the age of 50 years. Unemployed persons (76%) and those living in rural areas (53%) were more likely to report poor health. For individual social capital, persons with no trust (71%) and no civic participation (62%) reported poor health.

Table 4 presents the results of the multilevel regression analysis of factors associated with self-rated health, for the overall population. All the individual variables in Model 2, except for the individual social capital variables, were significantly related to self-rated health. Compared to males, females were less likely to report good health (Odds Ratio 0.82: Confidence Interval 0.73, 0.91). Marital status was negatively related to self-rated health, with unmarried persons less likely to report good health compared to their married counterparts. Increasing age was also negatively related to health (0.87: 0.85, 0.88).

**Table 4:** Multilevel regression analysis of factors associated with self-rated health.

Variables	Model 1	Model 2	Model 3	Model 4
	(Null)	(Individual variables)	(Community variables)	(Individual + community)
Gender				
Male		1		1
Female		0.82***(0.73, 0.91)		0.82***(0.73, 0.91)
Marital status				
Married/partners		1		1
Widow/divorced		0.84**(0.73, 0.97)		0.84**(0.73, 0.97)
Never married		0.87**(0.77, 0.99)		0.87**(0.77, 0.99)
Age		0.87*** (0.85, 0.88)		0.87***(0.85, 0.88)
Age squared		1.00***(1.00, 1.00)		1.00***(1.00, 1.00)
Education				
None		1		1
Primary		1.18**(1.03, 1.35)		1.18**(1.02, 1.35)
Secondary		2.07***(1.77, 2.42)		2.07***(1.78, 2.42)
Tertiary		3.74***(2.72, 5.15)		3.74***(2.72, 5.15)
Employment				
Unemployed		1		1
Employed		1.65***(1.47, 1.85)		1.64***(1.46, 1.85)
Household income quintile				
1		1		1
2		0.94 (0.81, 1.09)		0.94 (0.81, 1.09)
3		0.96 (0.83, 1.11)		0.96 (0.83, 1.11)
4		1.06 (0.91, 1.24)		1.06 (0.91, 1.24)
5		1.35***(1.13, 1.61)		1.34***(1.13, 1.60)
Government grants				
None		1		1
1 or more		1.39***(1.23, 1.56)		1.39***(1.23, 1.56)
Residence				
Urban		1		1
Rural		1.19**(1.05, 1.35)		1.20**(1.05, 1.36)
Smoking				
No		1		1
Yes		0.87**(0.77, 0.99)		0.87**(0.77, 0.99)
Individual social capital				
Trust				
No		1		1
Yes		1.00 (0.89, 1.11)		0.99 (0.89, 1.10)
Civic participation				
No		1		1
Yes		0.98 (0.89, 1.09)		0.98 (0.88, 1.08)
Neighbourhood social capital			1.09*(0.99, 1.19)	1.18***(1.06, 1.32)
Deprivation rank				
Most deprived			1	1
Moderately deprived			0.86* (0.71, 1.03)	0.92 (0.73, 1.16)
Least deprived			0.73***(0.61, 0.88)	0.81* (0.65, 1.03)
/lnsig2u	−1.94 (−2.31, −1.57)	−1.48 (−1.82, −1.13)	−2.06 (−2.45, −1.68)	−1.57 (−1.92, −1.22)
sigma_u	0.38 (0.31, 0.46)	0.48 (0.40, 0.57)	0.36 (0.29, 0.43)	0.46 (0.38, 0.54)
rho	0.04 (0.03, 0.06)	0.06 (0.05, 0.09)	0.04 (0.03, 0.05)	0.06 (0.04, 0.08)
BIC	13854	11024	13870	11042

*** p<0.01, ** p<0.05, * p<0.1.

Education was observed to be positively related to health status, with higher education associated with better health outcomes. Compared to those with no education, respondents with tertiary education were more likely to report good health status (OR 3.52: 95%CI 2.56, 4.86). Compared to married persons, the divorced (0.80: 0.71, 0.96) and single (0.85: 0.75, 0.96) were less likely to report good health. The likelihood of reporting good health increased with higher education. Compared to those with no education, individuals at tertiary level were more likely to report good health (3.74: 2.72, 5.15). Households in the highest quintile were more likely than those in lower quintiles to have a positive health outcome (1.35: 1.13, 1.61). Conversely, households which received government grants were highly likely to report good health (1.39: 1.23, 1.56). Living in a rural area increased the likelihood of reporting good health (1.19: 1.05, 1.35), while smoking was more likely to reduce a person's health status (0.87: 0.77, 0.99).

In Model 3, which controlled for neighbourhood social capital and deprivation index, there was a positive, but weak association between neighbourhood social capital and self-rated health (1.09: 0.99, 1.19). Neighbourhood economic deprivation was negatively associated with self-rated health, implying that the higher the neighbourhood deprivation score, the lower the odds of reporting good health.

In Model 4, when all the individual and community variables were included in the analysis, there was no change in the odds ratios of individual variables. Neighbourhood social capital remained positive, but its association with self-rated health was much stronger (1.18: 1.06, 1.32). The odds associated with deprivation rank were higher, but the strength of the association was weak.

Table 5 gives the results of the analysis with adjusted odds ratios, stratified by province. There were variations in the association between both individual and community variables. Education was positively related to self-rated health in all the provinces, but to a varied degree. In Mpumalanga, persons with tertiary education were 10 times more likely to report good health than non-educated individuals (10.0: 2.08, 48.12). This is compared to 3.35(1.80, 5.88) for North West and 6.48(2.67, 15.74) in Western Cape. Though not statistically significant, gender was positively associated with self-rated health in Western Cape, but this relationship was negative in most provinces. In Kwa Zulu Natal (0.61: 0.39, 0.96) and North West (0.63: 0.50, 0.79), this association was statistically significant, and females were less likely than males to report good health. Increasing age reduced the likelihood of reporting good health in all the provinces. In almost half of the provinces, employed persons were twice as likely as unemployed persons to report good health. Persons living in rural areas were more likely to report good health in most provinces, with statistically strong associations in Eastern Cape (1.96: 1.34, 2.86) and Western Cape (1.41: 0.94, 2.12). In Limpopo, however, persons living in rural areas were less likely to report good health. Households receiving government grants were more likely to report poor health in almost all but 1 province.

**Table 5:** Multilevel regression analysis of factors associated with self-rated health, by province (adjusted odds ratios).

Variables	Western Cape	Eastern Cape	Northern Cape	Free State	KwaZulu-Natal	North West	Gauteng	Mpumalanga	Limpopo
Gender									
Male	1	1	1	1	1	1	1	1	1
Female	1.13(0.84, 1.53)	0.74*(0.54, 1.02)	0.95(0.66, 1.38)	1.01(0.66, 1.53)	0.61**(0.39, 0.96)	0.63***(0.50, 0.79)	0.96(0.61, 1.51)	1.09(0.71, 1.67)	0.94(0.59, 1.49)
Marital status									
Single	1	1	1	1	1	1	1	1	1
Married/cohabiting	0.94(0.63, 1.40)	0.97(0.66, 1.43)	1.25(0.70, 2.25)	0.87(0.52, 1.46)	0.65(0.37, 1.15)	0.65***(0.50, 0.86)	1.23(0.68, 2.21)	0.60*(0.33, 1.10)	0.70(0.41, 1.19)
Divorced/widowed/separated	0.89(0.62, 1.28)	0.686**(0.48, 0.98)	0.88(0.55, 1.41)	0.47***(0.29, 0.77)	1.04(0.67, 1.61)	1.02(0.81, 1.28)	1.69**(1.01, 2.81)	0.70(0.44, 1.13)	0.71(0.40, 1.25)
Age	0.81***(0.77, 0.86)	0.87***(0.84, 0.91)	0.89***(0.83, 0.95)	0.84***(0.80, 0.90)	0.87***(0.82, 0.92)	0.87***(0.85, 0.90)	0.95(0.89, 1.02)	0.85***(0.80, 0.90)	0.85***(0.80, 0.90)
Age squared	1.00***(1.00, 1.00)	1.00***(1.00, 1.00)	1.00**(1.00, 1.00)	1.00***(1.00, 1.00)	1.00***(1.00, 1.00)	1.00***(1.00, 1.00)	1.00(1.00, 1.00)	1.00***(1.00, 1.00)	1.00***(1.00, 1.00)
Education									
None	1	1	1	1	1	1	1	1	1
Primary	1.26(0.79, 2.00)	1.11(0.77,1.62)	0.93(0.52, 1.66)	1.68(0.89, 3.16)	0.95(0.58, 1.57)	1.03(0.81, 1.31)	1.93(0.82, 4.53)	1.15(0.68, 1.97)	1.25(0.73, 2.14)
Secondary	1.93***(1.17,3.17)	2.27***(1.47, 3.50)	1.652(0.90, 3.04)	2.65***(1.34, 5.26)	1.63*(0.96, 2.78)	1.91***(1.44, 2.53)	3.49***(1.50, 8.14)	2.11**(1.15, 3.88)	2.30**(1.20, 4.41)
Tertiary	6.48***(2.67, 15.74)	5.46**(1.48, 20.18)	4.08**(1.04, 16.05)	2.76*(0.97, 7.86)	2.16(0.79, 5.91)	3.25***(1.80, 5.88)	2.13(0.65, 6.97)	10.0***(2.08, 48.12)	4.33*(0.85, 21.99)
Employment									
Unemployed	1	1	1	1	1	1	1	1	1
Employed	2.64***(1.92, 3.63)	2.05***(1.40, 3.02)	2.04***(1.33, 3.11)	1.52*(0.99, 2.34)	1.31(0.85, 2.02)	1.44***(1.15, 1.81)	1.31(0.84, 2.06)	1.54**(1.01, 2.36)	2.28***(1.30, 4.01)
Household income quintile									
1	1	1	1	1	1	1	1	1	1
2	0.68(0.33, 1.42)	0.85(0.56, 1.28)	1.10(0.56, 2.16)	1.11(0.57, 2.15)	1.21(0.67, 2.15)	1.01(0.79, 1.30)	0.87(0.42, 1.82)	1.20(0.64, 2.25)	0.64(0.38, 1.11)
3	0.87(0.43, 1.74)	0.84(0.56, 1.25)	1.19(0.63, 2.24)	0.96(0.51, 1.82)	1.39(0.75, 2.58)	0.81(0.62, 1.05)	1.31(0.64, 2.69)	0.93(0.50, 1.74)	0.78(0.41, 1.49)
4	0.70(0.36, 1.39)	0.70*(0.46, 1.07)	0.88(0.46, 1.66)	1.07(0.56, 2.05)	0.89(0.49, 1.63)	0.93(0.69, 1.26)	1.30(0.65, 2.58)	1.59(0.81, 3.15)	0.51**(0.27, 0.98)
5	1.31(0.60, 2.85)	1.31(0.63, 2.72)	0.74(0.33, 1.66)	1.41(0.59, 3.37)	1.40(0.63, 3.08)	1.82**(1.14, 2.90)	2.20*(0.98, 4.92)	1.06(0.46, 2.41)	0.47(0.16, 1.41)
Government grants									
None	1	1	1	1	1	1	1	1	1
1 or more	0.65***(0.48,−0.89)	1.19(0.84, 1.70)	0.70(0.45, 1.07)	0.81(0.53, 1.21)	0.63**(0.41, 0.98)	0.73**(0.57, 0.93)	0.50***(0.32, 0.76)	0.62**(0.39, 0.99)	0.98(0.57, 1.68)
Residence									
Urban	1	1	1	1	1	1	1	1	1
Rural	1.41*(0.94, 2.12)	1.96***(1.34, 2.86)	1.36(0.80, 2.31)	1.09(0.59, 2.03)	1.31(0.88, 1.94)	1.19(0.91, 1.55)	3.19(0.37, 27.80)	0.77(0.51, 1.17)	0.46**(0.24, 0.89)
Smoking									
No	1	1	1	1	1	1	1	1	1
Yes	0.83(0.62, 1.11)	1.10(0.76, 1.59)	0.78(0.53, 1.15)	0.79(0.49, 1.25)	0.51***(0.31, 0.84)	0.77*(0.58, 1.02)	1.33(0.79, 2.26)	1.00(0.58, 1.71)	1.42(0.78, 2.57)
Individual social capital									
Trust									
No	1	1	1	1	1	1	1	1	1
Yes	1.37*(0.98, 1.91)	1.01(0.71, 1.44)	0.77(0.49, 1.20)	0.70*(0.48, 1.02)	0.91(0.61, 1.36)	1.39***(1.13, 1.71)	0.78(0.52, 1.18)	1.04(0.71, 1.53)	0.56***(0.38, 0.84)
Civic participation									
No	1								
Yes	0.91(0.68, 1.22)	0.947(0.71, 1.26)	1.17(0.78, 1.75)	0.97(0.67, 1.41)	0.98(0.68, 1.42)	1.01(0.82, 1.25)	0.84(0.57, 1.23)	0.90(0.61, 1.31)	1.00(0.63, 1.59)
Neighbourhood social capital	0.60***(0.44, 0.82)	0.89(0.59, 1.33)	0.82(0.57, 1.17)	1.18(0.70, 1.98)	0.94(0.66, 1.34)	1.59***(1.27, 1.99)	1.90***(1.21, 2.97)	0.72**(0.55, 0.94)	1.06(0.86, 1.31)
Deprivation rank									
Most deprived	1	1	1	1	1	1	1	1	1
Moderately deprived	1.16(0.74, 1.81)	1.02(0.55, 1.88)	1.29(0.81, 2.06)	1.02(0.64, 1.62)	1.05(0.34, 3.22)	1.43(0.57, 3.56)	0.59*(0.33, 1.04)	1.54*(0.93, 2.55)	0.63(0.25, 1.62)
Least deprived	– ^#^	– ^#^	1.20(0.56, 2.55)	1.12(0.70, 1.80)	0.79(0.25, 2.53)	1.26(0.53, 3.03)	– ^#^	0.81(0.50, 1.32)	0.71(0.25, 2.01)

*** p<0.01, ** p<0.05, * p<0.1, ^#^none of the communities in this province were in the lowest deprivation rank.

There were variations in association as well as strength of the relationship between individual social capital and self-rated health among the provinces. In Western Cape (1.37: 0.98, 1.91) and North West (1.39: 1.13, 1.71), having trust increased the likelihood of reporting good health, while the reverse was true in Limpopo (0.56: 0.38, 0.84) and Free State (0.70: 0.48, 1.02). Civic participation did not have a statistically significant bearing on self-rated health.

Neighbourhood social capital also related differently to self-rated health between the provinces. In Western Cape (0.60: 0.44, 0.82) and Mpumalanga (0.72: 0.55, 0.94), neighbourhood social capital reduced the odds of reporting good health. In North West (1.59: 1.27, 1.99) and Gauteng (1.90: 1.21, 2.97), increased neighbourhood social capital improved the likelihood of reporting good health.

## Discussion

In this study, we used a multi-level analysis to examine the relationship between social capital and self-reported health status, while controlling for several factors. Two indicators of social capital were used: individual social capital, measured by two variables denoting trust and civic participation; and neighbourhood social capital, which was a composite measure denoting support, association, behaviour and safety in a community.

The individual indicators of social capital were not statistically significant predictors of self-rated health in the overall population. Thus we did not have sufficient evidence to conclude that trust and civic participation were related to health. This result is different from the findings of other studies, which relate social trust and civic participation to health [21], [31], [32]. Other studies, however, in conformity with our result, do not find an association between health and civic participation [16].

Despite there being no statistically significant association between individual social capital (civic participation) and health in the overall population in our study, we noted that when adjusted for other factors, both network participation and trust appeared to be negatively related to self-rated health, implying that the higher the individual social capital the lower the likelihood of reporting good health. Though the evidence is not sufficient to support this, our findings may seem to conform to literature that expounds the negative effects of social capital on individual health. Some types of participation may produce negative health behaviours, and also the same kinds of groupings and associations which can generate social capital may have the potential to exclude others [13]. Therefore, instead of fostering progress, social capital can become a constraint to individuals' actions and choices.

Our study, however, did show that when measured at the neighbourhood level, social capital was strongly related to self-rated health. Individuals in neighbourhoods with high social capital were more likely to report good health. This is similar to what has been found in other studies [33]. Neighbourhood social capital may lead to an improvement in health by increasing access to social amenities and services such as transportation, health and recreational facilities. Thus, even though the individual level social capital as measured by social trust and civic participation may move in the opposite direction, these measures may not reflect the benefits of living in a community that has a high social capital. As a result, the measures of social capital at individual level, as used in the literature, may not be sufficient to capture neighbourhood effects.

We disaggregated our analysis of social capital and health to the provincial level, and by so doing, we tried to study how context specific social capital impacts on individual health. The results show that there are variations in the way in which social capital influences health in the different provinces. In Western Cape and North West, social trust was positively related to health. In Limpopo and Free State, the opposite was true, in that social trust was negatively associated with health. In some instances, there was also a difference in how individual and neighbourhood social capital related to health in the same areas. For example, where as individual social capital was positively associated with health in Western Cape, neighbourhood level social capital moved in the opposite direction. The study, therefore, indicates that even within similar settings, social capital may have a different impact on health outcomes at different levels. It is therefore important in future analyses to take this into consideration and to delve deeper into understanding the mechanisms within which social capital influences health.

It could be expected that neighbourhood social capital from relatively affluent provinces such as the Western Cape will be positive. However, this was not always the case in our analysis, with neighbourhood social capital in Western Cape moving in the opposite direction. Gauteng and North West, however, had positive social capital. The discrepancy may be an indication of the poor distribution of resources in the Western Cape, and of the socio-economic inequities that still exist generally in the South African population, which negatively impact on health [34]. Our study therefore, makes a case for adopting interventions that will improve social capital at the neighbourhood level, by availing social amenities that will be accessible to all, and at the same time strengthening individual social capital by promoting bonding within the community. This has been shown to be achievable in South Africa [35].

Furthermore, there is need to tackle other socio-economic constraints that may have a negative impact on health. As has been shown in other studies [36], we found that females were more likely to report poor health. This could most probably be a result of their relatively low standing in society, which restricts access to health. This reinforces the need for gender based interventions aimed at improving health and reducing the social and economic gender inequalities in society [37]. Marriage is largely considered to be beneficial for health, with divorced and never married individuals displaying poor health status [38]. This was the same in our study, where we showed that compared to married persons, being single or divorced increased the likelihood of reporting poor health. Age was also shown to be negatively associated with health. Increasing age is generally associated with a decline in self-rated health [39]. In South Africa, the burden of non-communicable diseases, common with increasing age, is on the increase and exists side by side with the burden of infectious diseases [40]. This could account for the reports of poor health status among older individuals. There is, therefore, need for further investigations into the burden of disease, particularly co-morbidities, evidence of which is scarce in South Africa. Also noteworthy is the association between smoking and poor health. We found that smokers were more likely to report poor health. This has been shown to be the case in other studies [25], and there is thus need for concerted efforts to control smoking.

We found a positive association between indicators of economic standing and self-rated health. Compared to those with no education, persons with secondary and tertiary education were more likely to report good health. Similarly, employed persons and those in the highest income quintile were more likely to report good health. This is again reflective of the largely socio-economic disparities that persist in the South African society [34].

A major strength of this study was that we used data from a large nationally representative sample. However, the cross-sectional nature of the data limited our ability to draw causal inferences. While this study provides useful insight into the impact of social capital on health when taken in context, we cannot conclude on the basis of the results whether social capital is harmful or beneficial to health. We must take into consideration the fact that the association between social capital and health may vary depending on the nature of the health outcome and how it was measured. For instance, some illnesses such as depression may require a lot of psychosocial support at the individual level, and therefore factors such as trust and civic participation and family ties may be crucial to the improvement of health. Other conditions, mainly non-communicable diseases may be influenced by neighbourhood economic conditions. It is important therefore, to have a specific and objective measure of health. Thus, one limitation of this study was that the measure of health used was largely subjective and too general. However, self-rated health has been shown to be a good predictor of morbidity and mortality outcomes [1].

## Conclusion

Our study demonstrated the importance of considering contextual factors in the analysis of the relationship between social capital and health. We found that individual social capital as measured by social trust and civic participation was not significantly related to self-rated health. Neighbourhood social capital on the other hand was significantly associated with health. Analysis by province showed variations in the way in which social capital affected health in different contexts. We also showed the importance of other socio-economic factors such as education, age and gender in predicting self-rated health. We recommend the adoption of interventions that will improve neighbourhood social capital, and at the same time improve social ties. Further studies should be undertaken to understand the mechanisms of how social capital impacts on health in the South African population.
